# Effect of Recombinant Human Growth Hormone Treatment in a Patient with Short Stature Associated with the Ring Chromosome 17 Syndrome

**DOI:** 10.1155/2023/6686511

**Published:** 2023-09-25

**Authors:** Gustavo Tempone Cardoso Penna, Gabriela de Rezende Lelot, Ana Luiza de Rezende Lelot, Juliana Greghi Hernandez, Carolina Costa Figueiredo, Nara Michelle de Araujo Evangelista, Vania de Fatima Tonetto Fernandes, Guido de Paula Colares Neto

**Affiliations:** ^1^Centro Universitário São Camilo, Avenida Nazaré, 1501 CEP: 04263-200, São Paulo, SP, Brazil; ^2^Hospital Infantil Darcy Vargas, Rua Doutor Seráfico de Assis Carvalho, 34-Morumbi 05614-040, São Paulo, SP, Brazil

## Abstract

**Background:**

Ring chromosome 17 syndrome is a rare hereditary disorder whose prevalence is less than 1 : 1.000.000. We present a ten-year-old patient with ring chromosome 17 syndrome who had short stature and was treated with recombinant human growth hormone (rhGH). *Case Report*. A ten-year-old male scholar had moderate left conductive deafness, left kidney hypoplasia with hypertension, epilepsy, malformations in hands, feet, and abdomen, and disproportionately short stature. Despite no evidence of growth hormone deficiency, rhGH treatment was indicated as a therapeutic test due to his decelerated growth velocity and severe short stature. As a result, his growth velocity increased by 4.2 cm per year and his stature Z-score increased (from −5.87 to −5.23).

**Conclusion:**

The patient's severe short stature may be related to genetic, environmental, and hormonal factors and the positive response to rhGH may indicate abnormalities in the somatotropic axis that were mitigated with the treatment. Although rhGH associated with adequate comorbidities controls improved his growth velocity and height *Z*-score, its effects in the long term are still unclear.

## 1. Introduction

The ring chromosome syndrome is caused by the breakage of the distal portions of both chromosomes' arms and the subsequent fusion of its two ends. This abnormality can involve chromosomes such as 14, 17, and 20 and occur during meiosis or mitosis in the early embryo development, and it causes telomeric and genetic loss. Its phenotype depends on the number of genes lost, gene regulation, and mosaicism degree [1–3].

The ring chromosome 17 syndrome is a rare hereditary disorder whose prevalence is less than 1 : 1.000.000. When there is no involvement of Miller–Dieker's critical region, located in the short arm of chromosome 17 (17p13.3), a milder phenotype is presented with delayed growth and developmental milestones, reduced temporal lobes, epilepsy, flecked retina, cafe-au-lait spots, epicanthal folds, broad nasal bridge, and low-set ears. However, when it involves Miller–Dieker's region, the phenotype includes microcephaly, intellectual disability, hypotonus, opisthotonos, micrognathia, ear dysplasia, anteverted nostrils, and lissencephaly [3–5].

The present study aims to present a patient with ring chromosome 17 syndrome and severe short stature without the involvement of Miller–Dieker's critical region and its singular response to recombinant human growth hormone (rhGH) treatment.

## 2. Case Report

Informed consent for a case report was obtained.

PHLS is a ten-year-old boy born via C-section due to his mother's preeclampsia at 35 weeks of gestational age. According to Intergrowth-21st growth curves, he was smaller than average for his gestational age, with a birth weight of 1905 g (*Z*-score: −1.41), a birth length of 42 cm (*Z*-score: −2.01), and a birth head circumference of 31 cm (*Z*-score: −0.8). His parents are nonconsanguineous.

**Figure 1:**
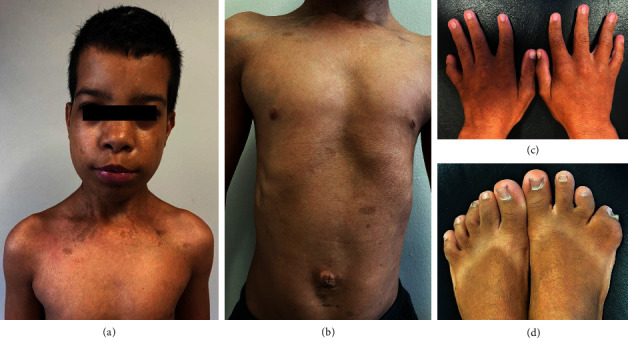
(a) Wide nasal bridge, epicanthal folds, low-set ears, and cafe-au-lait spots. (b) Umbilical hernia, diastasis recti, and cafe-au-lait spots. (c) Fifth left finger brachydactyly and bilateral clinodactyly of first and fifth fingers. (d) Bilateral clinodactyly of third toes and fourth and fifth right toes syndactyly.

Since birth, he presented a broad nasal bridge, epicanthal folds, low-set ears, fifth left finger brachydactyly, bilateral clinodactyly of first and fifth fingers and third toes, and fourth and fifth right toes syndactyly. Also, since birth, he had bilateral cryptorchidism, umbilical hernia, and diastasis recti, which were fixed surgically at ten and seven months (Figure 1). During his first two years, he evolved with delayed developmental milestones, moderate left conductive deafness, cafe-au-lait spots, and reduced growth velocity. Moreover, he was diagnosed with epilepsy after vaccination at six months old. He used phenobarbital regularly until he was nine, when it was suspended due to the absence of new seizures. Also, he had left kidney hypoplasia with consequent hypertension, which was diagnosed at the age of nine and treated with 2.5 mg amlodipine. There were no relevant chronic diseases or similar cases in the family, and his parents had no abnormalities in their karyotype.

At ten years and four months of age, his G band karyotype revealed a ring 17 chromosome (46 XY. r (17) (p13q25)), and he was referred to a pediatric endocrinology service to investigate his short stature. At that time, he was prepubertal; according to the World Health Organization (WHO)'s growth charts, he presented severely disproportionate short stature as his height *Z*-score was −5.64, i.e., below his familial growth channel (mid parental height *Z*-score: −0.02), slightly reduced arm span to height ratio (0.97), and eutrophy with a body mass index *Z*-score of −0.94.

Moreover, he had a neuropsychomotor development delay at that time, and the neurology department followed him without any prescribed medications. He maintained the use of 5 mg amlodipine due to hypertension, which was controlled.

At eleven years of age, screening laboratory tests did not present changes concerning the liver and kidney function, osteometabolic profile, thyroid function, or blood cortisol levels. Also, at that age, the X-ray revealed bone age compatible with chronological age, an undefined distal phalanx on his left fifth finger, and an extra numeracy right toe between the fourth and fifth toes.

At eleven and fourteen years of age, regarding the somatotropic axis, insulin-like growth factor-1 (IGF-1) and insulin-like growth factor binding protein 3 (IGFBP-3) levels were normal. Also, at fourteen years of age, the growth hormone (GH) stimulation test with clonidine was responsive with a peak GH level of 10.20 ng/ml at 90 minutes (reference >5 ng/ml). Moreover, magnetic resonance imaging showed no alterations in the hypothalamic-pituitary region at that age.

Due to his slow growth velocity, rhGH treatment was started with 0.10 UI/kg/day (0.033 mg/kg/day) as a therapeutic test at 14 years old and five months. After detecting no adverse effects, the dosage was increased to 0.15 UI/kg/day (0.045 mg/kg/day) and maintained as the usual dosage with IGF-1 and IGFBP-3 levels in their reference range. As a result of 16 months of treatment, his growth velocity increased from 3.5 cm per year to 7.7 cm per year, and his stature *Z*-score increased (from −5.87 to −5.23) according to the WHO's growth charts. During this period, he had no treatment complications.

Currently, at fifteen and nine months of age, he presents a delayed bone age compared to chronological (bone age: 13 years and chronological age: 15 years), he is pubertal with Tanner and Marshall staging G2P3. Nowadays, he maintains follow-ups with endocrinology, neurology, speech therapy, and nephrology without any complications or new symptoms, and he is still using 5 mg amlodipine, which is being reduced due to his blood pressure stabilization.

## 3. Discussion

This case report presents new findings that contribute to understanding the potential therapeutic effect of growth hormone in a patient with short stature associated with the ring chromosome 17 syndrome.

The ring chromosome 17 syndrome is a rare chromosomal condition described in 1971, with only 20 other cases reported in the literature, and 14 of those not associated with the Miller–Dicker syndrome [5, 6].

There are two main theories regarding the ring chromosome's formation. First, the DNA breakage in both subtelomeric regions causes the arms to merge, losing telomeres and distal chromosomal genetic material, the primary mechanism related to the 17 chromosomes. The second one is a telomere maintenance dysfunction, resulting in the shortening and merging of both terminal portions. Both can occur in gametogenesis, especially paternal, and in the mitosis of embryonic cells related to mosaicism. Moreover, the embryonic age determines the severity of the patient's phenotype [2, 3].

The ring chromosome 17 syndrome's phenotype is related to deletion and gene expression. Surace et al. described that telomere loss affects the expression of nearest genes due to the “telomere position effect” (TPE) phenomenon, in which subtelomeric regions show more epigenetic plasticity, with gains or losses of the gene function. Besides, genes located in the break spot may lose or depart from their regulatory regions. Thus, the phenotype depends on the genes involved in the deletion or shortening of the telomeres, TPE, and gene expression regulation [1, 2, 6].

Furthermore, ring chromosomes are mitotically unstable with secondary alterations in the cell division process, such as monosomies and double rings. Therefore, there are higher chances of mosaicism, in which the phenotype depends on the mosaicism's rate and cellular distribution in different tissues [1, 3].

This case report describes a nonmosaic patient, without supernumerary chromosomes, with one ring chromosome 17 linked by the portions p13 and p25 of its long and short arms. Since the ring chromosome 17 syndrome's phenotype is related to the preservation of Miller–Dicker's critical region (17p13.3), the patient's milder phenotype suggests that this critical region is partially or entirely preserved [4–6].

Deleting Miller–Dicker's critical region results in a more severe phenotype with lissencephaly, multiple craniofacial malformations, delayed developmental milestones, and reduced life span. The patient did not have any of these characteristics but had classic ring chromosome 17 syndrome characteristics such as mildly delayed developmental milestones, short stature, epilepsy, craniofacial malformations, and cafe-au-lait spots. He also presented some less common characteristics such as left deafness, brachydactyly, clinodactyly, syndactyly, left kidney hypoplasia, bilateral cryptorchidism, and some abdominal wall malformations such as umbilical hernia and diastasis recti [4, 5].

One of the patient's main characteristics was his disproportionately short stature (height Z-score below three and reduced arm span/height ratio), which may suggest skeletal abnormalities contributing to his short phenotype. Moreover, his short stature was aggravated by his comorbidities, such as left kidney hypoplasia, hypertension, epilepsy, long-term medications use, and surgical interventions. They may have impaired his growth velocity during critical height gain phases such as early infancy and prepuberty.

Environmental, hormonal, and genetic factors influence a child's stature. Bone and growth plate abnormalities are factors that significantly decrease height gain. Chronic diseases and their treatments, such as epilepsy with anticonvulsant drug use, can directly affect bone accrual and elongation and reduce final stature. Also, they may impair the somatotropic axis by promoting suboptimal GH secretion or impairing IGF-1-IGFBP-3 action continuously or transiently [7].

Notably, the integrity of the somatotropic axis guarantees an adequate child's growth, as GH and IGF-1 are critical regulators of longitudinal bone growth and skeletal maturation throughout life. So, laboratory and radiology evaluations are needed in growth disorders, but the current methods have limitations. For example, although IGF-1 serum concentrations show slight circadian variation, their levels can be reduced in undernutrition and chronic diseases or normal in growth hormone deficiency. Moreover, GH stimulation tests may not have a great sensibility to identify partial growth deficiency or GH resistance or may fail to respond in healthy peripubertal and obese children. These inaccuracies occur because the stimuli are not physiological and do not replicate normal secretory dynamics. Also, the periodic secretion of somatostatin might influence the somatotroph response [8, 9].

Despite no anatomical abnormalities in the hypothalamic-pituitary region or laboratory evidence of growth hormone deficiency in the patient's diagnostic workup, rhGH treatment was indicated as a therapeutic test due to the absence of previous reports of rhGH treatment in ring chromosome 17 syndrome and the presence of comorbidities that could impair the somatotropic axis. In addition, the higher dose of rhGH was intended to overlap a possible GH resistance. As a result, the rhGH treatment improved his growth velocity and height Z-score. This positive effect may indicate abnormalities in the somatotropic axis, such as GH resistance, impaired IGF-1-IGFBP-3 actions, or a partial growth deficiency that was mitigated using rhGH. Also, the comorbidities control and the onset of puberty probably contributed to his height improvement [7, 10].

While this case report provides valuable insights into the potential therapeutic use of rhGH in a patient with ring chromosome 17 syndrome and severe short stature, it is essential to acknowledge certain limitations. First, the rarity of ring chromosome 17 syndrome inherently limits the generalizability of the findings to a broader population. In addition, the observed positive response to rhGH treatment raises intriguing questions about other underlying genetic and hormonal factors contributing to the patient's growth impairment. Moreover, this case report does not provide conclusive evidence regarding the long-term effectiveness of rhGH in individuals with this syndrome. Despite these limitations, this case report underscores the importance of meticulous documentation and exploration of rare genetic conditions, as it may shed light on novel treatment approaches and enhance our understanding of the underlying mechanisms involved.

In conclusion, although the patient had a satisfactory response to rhGH, careful surveillance is mandatory to understand the medication benefits and possible effects in the long term in the ring chromosome 17 syndrome.

## Data Availability

All data generated or analysed during this study are included within the article.
